# Influence of biological sex and training history on force production and its variability in humans

**DOI:** 10.3389/fphys.2026.1783010

**Published:** 2026-07-13

**Authors:** Edoardo Lecce, Paolo Amoruso, Fiorella Martire, Alessandro Scotto di Palumbo, Massimo Sacchetti, Ilenia Bazzucchi

**Affiliations:** Laboratory of Exercise Physiology, Department of Movement, Human and Health Sciences, University of Rome ‘Foro Italico’, Rome, Italy

**Keywords:** ballistic contractions, conduction velocity, EMG, muscle force, RFD, sex differences, strength

## Abstract

**Introduction:**

Maximal and rapid force production represent the principal metrics of neuromuscular performance, yet both show substantial inter-individual and day-to-day variability. Biological sex and training history are known to considerably affect the absolute magnitude of these measures; however, their influence on variability is unclear.

**Methods:**

To address this gap, we examined twenty-two young adults (12 males, 10 females), balanced for strength-trained (ST) and recreationally active (RA) status, who performed maximal ballistic isometric elbow-flexion trials in two sessions separated by 48–72 h. We assessed *maximal voluntary force* (MVF), *rate of force development* (RFD), and impulse during the initial 250 ms from force onset, and estimated maximal muscle-fiber conduction velocity (MFCV_MAX_) from high-density EMG over the same window. Day-to-day variability was computed for all these metrics.

**Results:**

Both sex and training history significantly affected absolute MVF, RFD, and impulse (*p* < 0.05). In contrast, MFCV_MAX_ differed only by training history, being 0.14 m·s^−1^ higher in ST (*p* < 0.05). ST participants also showed lower between-day variability in RFD and MFCV_MAX_ than RA (*p<*0.05). However, no differences were found for sex. Maximal RFD and impulse strongly correlated with MFCV_MAX_ across all groups (*R²*>0.6, *p* < 0.01).

**Discussion:**

Because MFCV_MAX_ is considered an indirect marker of the recruitment of larger motor units, our results suggest that ST participants may recruit relatively larger motor units during the initial contraction phase compared with RA individuals. In addition, more stable RFD across days is associated with comparable contribution from similarly sized motor units. Thus, within the present sample, day-to-day stability in rapid force appeared to be more strongly associated with training-related consistency than with biological sex.

## 1 Introduction

Neuromuscular performance is typically quantified by *maximal voluntary force* (MVF) and *rate of force development* (RFD) in humans during isometric muscle actions (Maffiuletti et al., 2016; Folland et al., 2014; Lecce et al., 2025c; Taber et al., 2016; Lecce et al., 2026). MVF represents the maximal force produced during a voluntary contraction and primarily reflects the maximal neural drive transmitted by spinal motoneurons to muscles and their contractile properties (Lecce et al., 2026). On the other hand, RFD is strongly associated with the level of neural drive and motor unit recruitment rate within the initial phase (~ 50 ms) of a contraction (Maffiuletti et al., 2016; Miyamoto et al., 2020; Del Vecchio, 2023), with contractile properties, such as fiber type and myosin heavy chain composition, muscle cross-sectional area, and the visco-elastic properties of the muscle–tendon complex, contributing more prominently after ~ 100 ms from contraction onset (Folland et al., 2014; Maffiuletti et al., 2016; D’Emanuele et al., 2023). Both metrics present substantial between-individual and day-to-day variability (Allen et al., 1995; Maffiuletti et al., 2016; Del Vecchio et al., 2019; Lecce et al., 2025c), but RFD appears to fluctuate considerably more than MVF (Morton et al., 2005; Pereira et al., 2011; Knaier et al., 2019; Levernier and Laffaye, 2021; Oranchuk et al., 2022). Evidence suggests diurnal rather than between-day variability (Lagerquist et al., 2006; Tamm et al., 2009; Hirono et al., 2024). Biological sex and training history emerge as leading factors affecting the absolute levels of maximal and rapid force production (Lecce et al., 2026).

Sex differences in neuromuscular performance are well established in humans (Lulic-Kuryllo and Inglis, 2022; Hunter and Senefeld, 2024; Lecce et al., 2024a; Joyner et al., 2025). Young male individuals typically exhibit higher MVF and RFD than female individuals (Glenmark et al., 2004; Inglis et al., 2013; Salvaggio et al., 2025), differences largely attributed to muscle-fiber size and composition that maximize force-generating capacity (Hunter et al., 2023; James et al., 2025; Lecce et al., 2026). However, when rapid force production is expressed relative to maximal force capacity (i.e., normalized to MVF), these sex differences are substantially reduced or absent under specific conditions, such as during rapid isometric contractions of the knee extensors across a range of submaximal-to-maximal efforts (Salvaggio et al., 2025). These observations are accompanied by similar normalized twitch and EMG amplitudes (Hannah et al., 2012) and a comparable maximal discharge rate of motor units (Grootenhuis et al., 2025), implying that *maximal strength* accounts for most of the sex-related disparities in rapid force production. However, evidence on day-to-day variability in maximal and rapid force production is contrasting: some reports describe greater consistency in female individuals (Luger et al., 2020), whereas others report no differences (Dutra et al., 2023), leaving no precise consensus or physiological determinants in this context.

Training history likewise exerts a profound influence on neuromuscular performance. Chronically strength-trained individuals generally present higher MVF and RFD and less relative variability than untrained or recreationally active counterparts (Lisee et al., 2019; Maden-Wilkinson et al., 2020; Levernier and Laffaye, 2021; Rizzato et al., 2024; Trybulski et al., 2025). These disparities have been attributed to greater EMG amplitude (Tillin et al., 2010) and a higher motor-unit discharge rate during early contraction phases (Škarabot et al., 2024). However, when force output is normalized to the MVF, these neural characteristics do not explain the mechanical output, suggesting that contractile properties are plausibly the leading parameter underlying differences in absolute force between strength-trained and untrained individuals (Casolo et al., 2021; Škarabot et al., 2024). Notably, earlier recruitment of higher-threshold motor units (those with larger fiber diameters) better explains variability in peak force and force rise-time during rapid contractions (Totosy De Zepetnek et al., 1992; Heckman and Enoka, 2012; Del Vecchio, 2023).

To date, direct decomposition of large motor-unit populations during *maximal voluntary contractions* (MVCs) to quantify effective neural drive while simultaneously assessing MVF and maximal RFD presents methodological limitations (Del Vecchio et al., 2020; Grison et al., 2025). An established index of recruitment is *muscle-fiber conduction velocity* (MFCV), which correlates with both absolute force and RFD (Methenitis et al., 2016; Del Vecchio et al., 2017, 2018b) and is closely associated with muscle-fiber size (Hakansson, 1956; Andreassen and Arendt-Nielsen, 1987; Masuda and De Luca, 1991; Del Vecchio et al., 2018c; Casolo et al., 2023). Accordingly, strength-trained athletes exhibit higher MFCV than untrained or recreationally active individuals during evoked and voluntary isometric contractions (Methenitis et al., 2016; Del Vecchio et al., 2018b). By contrast, males display higher MFCV than females during evoked quadriceps contractions from rest (Mase et al., 2006; Kouyoumdjian and Graca, 2023), but no sex differences emerge during isometric MVCs (Inglis et al., 2017), leaving uncertainties regarding possible similarities in the recruitment patterns during ballistic contractions.

Despite this literature, no consensus exists on the neuromuscular determinants underlying day-to-day variability in MVF and RFD, nor on the influence of biological sex and training history on these underlying mechanisms. To address these gaps, we recorded high-density EMG (HDsEMG) from the elbow flexors to estimate MFCV during voluntary ballistic contractions in a mixed-sex sample with differing training histories across two separate sessions. Importantly, MFCV can be reliably estimated from HDsEMG in very short time windows (~ 25 ms), enabling inference of recruitment patterns during ballistic isometric contractions (Farina et al., 2001; Del Vecchio et al., 2018a), with high reproducibility (Farina et al., 2004b; Casolo et al., 2020).

The primary aim was to determine whether (i) biological sex and (ii) training history influence the absolute magnitude and day-to-day variability of MVF and maximal RFD, and whether these metrics would parallel distinct neuromuscular characteristics assessed with *maximal MFCV* (MFCV_MAX_).

Based on the abovementioned evidence, we hypothesized that: (a) male individuals will exhibit greater MVF and maximal absolute RFD than female individuals, but similar relative RFD; (b) chronically strength-trained participants will show higher MVF and RFD with reduced variability between the two testing sessions (i.e., day-to-day variability); and (c) these differences will be accompanied by higher and more consistent MFCV_MAX_ between sessions.

## 2 Methods

### 2.1 Participants and ethical approval

The study was approved by the local ethics committee of the *Foro Italico* University of Rome (approval *CAR 231/2025*) and adhered to the standards of the *Declaration of Helsinki*.

Sample size was determined *a priori* using G*Power 3.1 (Faul et al., 2007), for the primary dependent variable (i.e., RFD) with the aim of detecting the expected group × time interaction in the two-group, two-session design. The following parameters were set: *f* = 0.3, *α* = 0.05, 1*-β* = 0.8, two groups with two time points, *r* = 0.6. The estimated sample size required for the present investigation was 20 (10 per group). We included 22 participants (females, n=10; males, n=12) to account for a potential ~ 10% dropout rate.

All participants provided written informed consent after receiving a detailed explanation of the experimental procedures, potential risks, and their right to withdraw from the study at any time without consequences. To ensure confidentiality, each participant was assigned a unique alphanumeric code. Inclusion criteria required participants to be between 18 and 35 years old, in good health, and classified as either *strength-trained* (ST) or *recreationally active* (RA) based on their training history (see study overview). Exclusion criteria included metabolic disorders, upper limb musculoskeletal disorders, acute infections, uncontrolled hypertension, use of medications affecting muscle protein metabolism, vascular tone, or neural activity, and use of oral contraceptives due to their direct impact on neuromuscular performance (Burrows and Peters, 2007; Elliott-Sale et al., 2020; Reif et al., 2021; Lecce et al., 2024a).

### 2.2 Study overview

Prior to the first experimental session, volunteers were screened for their habitual physical activity using the *International Physical Activity Questionnaire* [IPAQ] (Craig et al., 2003). ST volunteers were required to have engaged in a strength-training (not power) program targeting the upper limbs for a minimum of 3 years and at least 3 times per week, as outlined in updated ACSM guidelines (American College of Sports Medicine, 2009; Bishop et al., 2025). RA volunteers were required to engage only in habitual light-to-moderate aerobic physical activity (e.g., walking, cycling, recreational jogging) fewer than 2 times per week and to have no history of regular resistance, strength, or power training in the previous 3 years (McKay et al., 2022).

Participants visited the laboratory on three occasions. The first visit involved a familiarization with the experimental protocol, which included ballistic contractions of the dominant elbow flexor muscles, during which volunteers were instructed to reach their maximal force *as fast as possible*. The elbow flexors were chosen in this study as they have been shown to provide estimates of MFCV with high reliability (Farina et al., 2004b; Del Vecchio et al., 2018b). Limb dominance was assessed using the *Edinburgh Handedness Inventory Questionnaire* (Oldfield, 1971), and no measurements were performed during this first visit. The second and third visits involved the same experimental testing, separated by 48–72 h to minimize residual neuromuscular fatigue while preserving comparable physiological conditions across assessments (Knaier et al., 2019). On each visit, participants performed MVCs and maximal ballistic contractions with concurrent HDsEMG recordings. Participants completed all testing sessions at approximately the same time of day (± 1 h) to minimize potential diurnal variation in neuromuscular performance (Racinais et al., 2005).

Female participants were tested during either the ovulatory or mid-luteal phase to minimize fluctuations in neuromuscular performance and to ensure a comparable hormonal milieu across participants (Tenan et al., 2013; Weidauer et al., 2020; Piasecki et al., 2023). These phases were selected because neuromuscular characteristics are generally comparable and, in contrast to the early follicular phase, where greater variability in corticospinal excitability has been reported, provide more stable conditions (Piasecki et al., 2024). Menstrual phases were determined using the validated *Menstrual Practices Questionnaire* (Hennegan et al., 2020, 2022; Vagha et al., 2023; Wihdaturrahmah and Chuemchit, 2023), with day 1 identified as the onset of bleeding and forward counting used to identify the testing window. Participants reported the onset and duration of their two previous cycles to confirm regularity and improve phase estimation accuracy.

All participants were asked to avoid strenuous exercise for 48 h and caffeine consumption for 24 h prior to testing (Bazzucchi et al., 2011; Behrens et al., 2023).

### 2.3 Experimental design

**Figure 1:**
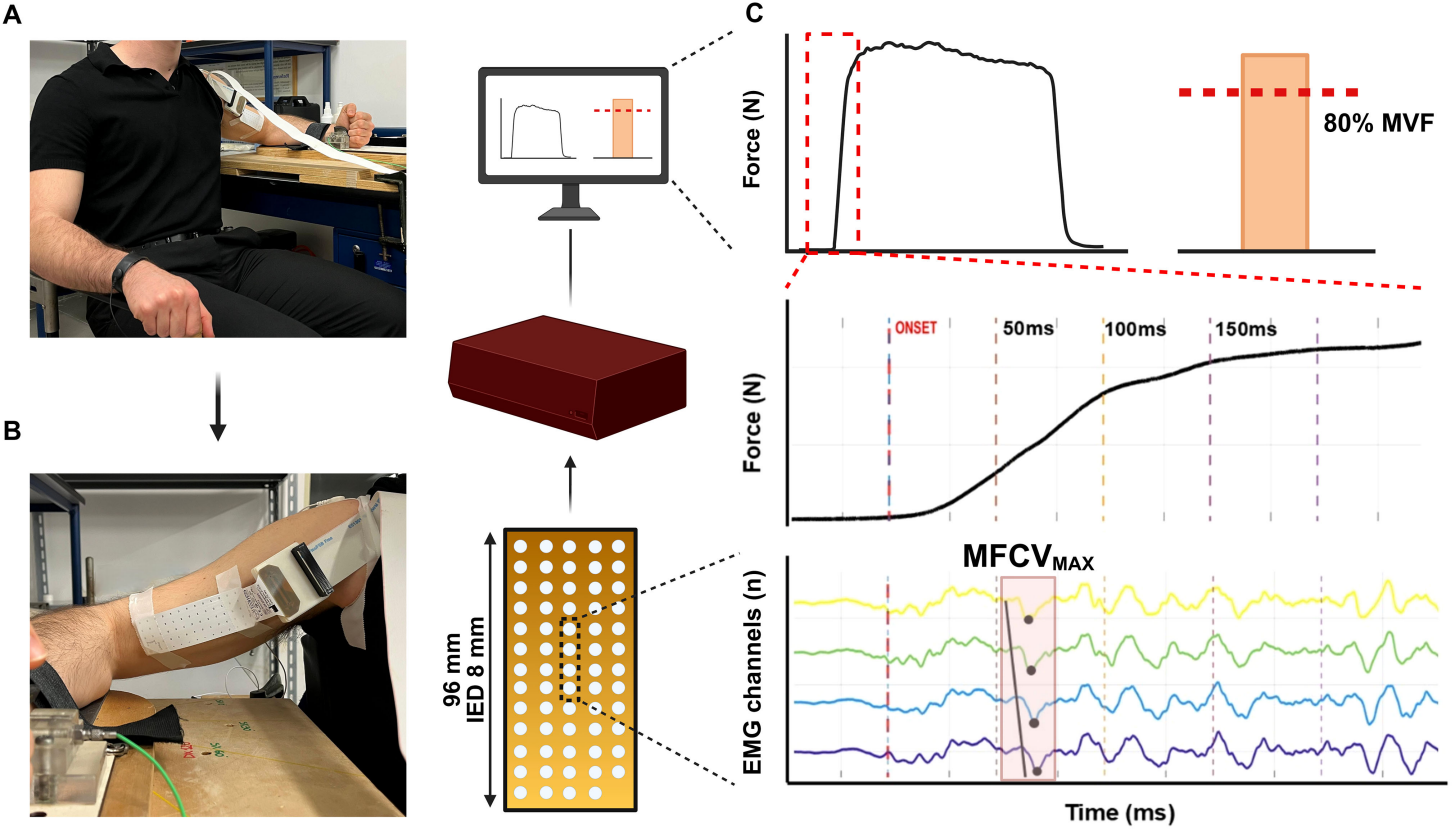
Experimental setup and analysis. **(A)** Participant positioned for the experimental session with a monitor placed 1.5 m from their eyes displaying their live force trace and a horizontal cursor with the magnitude of force produced. **(B)** Bidimensional HDsEMG grid (13 rows x 5 columns; inter-electrode distance [IED] = 8 mm) application onto the biceps brachii belly (elbow-flexor belly) with the connector to the EMG amplifier, synchronized with the force signal. **(C)** The force and the EMG signals were recorded during the maximal ballistic task, consisting of exceeding a threshold of 80% MVF *as fast as possible*. The force signal was assessed in time-locked windows of 50 ms from the onset to 150 ms, while MFCV_MAX_ was estimated through the action potential delay identified from the selected EMG channels (4 central channels as an example in the figure). The image was created with *BioRender*.

Before each session, participants completed a standardized warm-up consisting of 8 isometric submaximal elbow flexor contractions (4 × 50%, 3 × 70%, 1 × 90% of their perceived MVF), each separated by 30 s. After the warm-up, participants rested for 5 minutes before performing three elbow flexor MVCs, separated by 180 s, and were instructed to push *as hard as possible*. The highest force value was used to set the MVF. 5 min after the last MVC, participants performed five maximal isometric ballistic contractions of ~ 3–5 s duration. They were instructed to relax and flex the elbow *as fast and hard as possible*, with strong verbal encouragement during each trial (McNair et al., 1996) and a recovery period of 180 s. Volunteers were instructed to produce maximal force ‘*as fast as possible’* by exceeding the 80% MVF of the daily MVF set as a threshold (**Figure 1**), which was displayed with a horizontal cursor on the monitor positioned 1.5 m from their eyes as previously indicated (Del Vecchio et al., 2019; Škarabot et al., 2024).

### 2.4 Force and HDsEMG recording

Both familiarization and experimental sessions were conducted with the participant comfortably seated and the tested arm fixed to a custom rigid support for elbow flexors, which had previously been used for neuromuscular assessments (Menotti et al., 2012). The arm was abducted to 90°, the elbow flexed to 90°, and the forearm held in supination so that the anterior wrist abutted a flat metal plate attached and perpendicular to a force transducer (Model 9203; Kistler, Winterthur, Switzerland). The chair configuration was established during the familiarization, adjusted to each participant’s anthropometric characteristics, and replicated in the experimental sessions. Two waist straps were fastened across the pelvis, and the elbow joint was secured in a padded brace with Velcro straps. This setup was comfortable and well tolerated by the study participants. This arrangement enabled isometric elbow-flexor contractions in the horizontal plane (Menotti et al., 2012).

The analogue force signal was amplified using a charge amplifier (× 1000; Type 5011; Kistler) and sampled at 2048 Hz via an external analogue-to-digital converter (EMG-Quattrocento; OT Bioelettronica, Turin, Italy). Participants received real-time visual feedback of the force trace using the acquisition software (OTbiolab; OT Bioelettronica).

For the HDsEMG recordings, a bidimensional grid of 64 electrodes (13 rows × 5 columns; gold-coated; electrode diameter 1 mm; inter-electrode distance [IED] 8 mm; GR08MM1305, OT-Bioelettronica) was used. After shaving, light abrasion, and cleansing with 70% ethanol, the elbow flexor perimeter (i.e., the biceps brachii muscle belly) was identified by palpation and marked with a surgical pen. Grid orientation was determined from preliminary recordings with a 16-electrode array (IED 5 mm; OT-Bioelettronica) to locate the innervation zone (IZ) and estimate fiber direction (Del Vecchio et al., 2017; Lecce et al., 2025a). The IZ was identified as the inversion point of action-potential propagation along an electrode column (proximal-distal), and the HDsEMG grid was centered over the IZ (**Figure 1**). The high number of electrodes employed enables more accurate selection of channels based on the propagation of action potentials, thereby improving the reliability of MFCV estimates considerably compared to a limited number of channels (Farina et al., 2004b; Staudenmann et al., 2005).

The HDsEMG grid was positioned over the muscle belly, with a disposable bi-adhesive perforated foam layer (SpesMedica, Genoa, Italy) adapted for the grid. Adhesive holes were filled with conductive paste (SpesMedica) to ensure skin-electrode contact. To ensure consistent placement across sessions, anatomical landmarks and skin marks were traced onto acetate templates during the first assessment (Orssatto et al., 2023; Lecce et al., 2025a). A ground electrode was placed on the contralateral ulnar styloid process, and the reference electrode was positioned on the ipsilateral acromion.

The HDsEMG signals were acquired in monopolar mode, sampled at 2048 Hz, amplified (× 150) and band-pass filtered (10–500 Hz) to remove direct-current offset and aliasing artifacts. Signals were digitized with a multichannel amplifier at 16-bit resolution (3 dB bandwidth 10–500 Hz; EMG-Quattrocento; OT-Bioelettronica) and synchronized with the force trace from the same acquisition system for subsequent offline analysis.

### 2.5 Data processing

*Force signal analysis*: In the offline phase, the analog force signal was converted into *Newtons* (N). We then removed the contractions that showed pre-tension or countermovement, which was assessed as changes in baseline force of 0.5 N within the 150 ms before force onset (Škarabot et al., 2024). A zero-lag low-pass filter with a cut-off frequency of 400 Hz was applied to the whole length of the force signal. This large bandwidth is necessary for high accuracy when visually determining the force onset (Tillin et al., 2013), which was subsequently identified by an experienced investigator using previously described criteria (Tillin et al., 2010). Briefly, the force signal was first viewed with a y-axis scale of ~1 N and an x-axis scale of 500 ms to establish baseline noise. Onset was defined as the last peak or trough before a clear deflection from baseline. The cursor was then verified at higher resolution (y-axis: ~0.5 N; x-axis: 25 ms) and confirmed by simultaneously displaying the first derivative of the force-time trace (**Figure 1**). This approach has been demonstrated to be highly reliable and provide more accurate onset identification than automated algorithms (Tillin et al., 2010, 2013; Maffiuletti et al., 2016).

After onset identification, the force signal was low-pass filtered with a 20-Hz zero-lag third-order Butterworth filter, which eliminates high-frequency noise from the load cell and ensures an undistorted force output relative to the original signal (Del Vecchio et al., 2018b, 2019). The best trial was selected based on the following criteria: (*i*) the highest force produced at 100 ms following force onset, (*ii*) displaying no countermovement or pre-tension (< 0.5 N), and (*iii*) exhibiting sufficient force output (> 80% MVF) (Škarabot et al., 2024; Grootenhuis et al., 2025). The chosen force traces were analyzed over the 250 ms following force onset.

For this interval, the first derivative of force (i.e., RFD) was computed for overlapping windows from onset to X ms, where X varied from 1 to 250 ms, to identify the maximal RFD (RFD0-X_MAX_), which is defined as the peak force derivative (Del Vecchio et al., 2024). The time at which RFD0-X_MAX_ occurred (t-RFD_MAX_) was also recorded for each participant to assess potential group differences in the temporal profile of rapid force development. RFD was also computed for fixed windows up to 150 ms (0–50 ms, 50–100 ms, and 100–150 ms [**Figure 1]**) because most changes in RFD during isometric ballistic contractions occur before ~ 150 ms from force onset (Maffiuletti et al., 2016; Kozinc et al., 2022; Del Vecchio, 2023). The impulse (integral of the force-time curve) was also computed from force onset to 250 ms and thereby reflected the time history of the performed contraction within this time window. Because impulse is proportional to change in *momentum* (mass × change in velocity), it relates directly to elbow flexor speed when the wrist is not restrained (Aagaard et al., 2002; Del Vecchio et al., 2019). In addition to *absolute* explosive force measures (RFD, impulse), *relative* indices (RFD and impulse normalized to MVF) were computed to assess the participants’ ability to rapidly express their available force capacity during the rising phase of ballistic contractions (Folland et al., 2014; Lecce et al., 2025c). All offline analyses were performed using MATLAB 2022 (MathWorks Inc., Natick, MA, USA).

*EMG signal analysis*: Single-differential HDsEMG signals were calculated from the monopolar derivations for each column of the bidimensional array. Single-differential HDsEMG signals for each column were visually inspected, and a minimum of four single-differential HDsEMG channels with the highest *coefficient of correlation* (CC) and clear motor unit action potential propagation without shape change from the nearest innervation zone to the distal tendon were chosen for the analysis, with a cut-off of CC ≥ 0.85, discarding those displaying lower CC values. The grid columns selected for the MFCV estimates corresponded to the three central columns of the two bidimensional arrays, which corresponded to the channels with the highest quality (CC and propagation).

MFCV was computed using an algorithm that allows highly accurate estimates of conduction velocities from multichannel EMG and whose reliability and validity have been previously assessed in controlled and ballistic isometric contractions (Farina et al., 2001, 2004a, 2004b; Del Vecchio et al., 2018b) with a robust reliability as indicated by excellent ICC (≥ 0.88) (Martinez-Valdes et al., 2016). The use of ≥ 4 EMG channels allowed us to detect changes in MFCV as small as 0.1 m·s^-1^ compared with estimates from a pair of bipolar signals (0.4 m·s^-1^) (Farina et al., 2002). The same channels were used to compute the MFCV across sessions to guarantee an analogous signal comparison. MFCV_MAX_ was estimated between 100 ms before the force onset and 250 ms after the force onset as the highest identified velocity according to the level of CC (i.e., the highest level of CC was retained to compute the delay estimation) using a validated approach (Farina et al., 2004b; Pozzo et al., 2004; Del Vecchio et al., 2018b). The choice of this interval for estimating MFCV_MAX_ was based on accounting for both the delay between motor unit recruitment and their relative twitch force during the rising phase, and the physiological *electromechanical delay* (Del Vecchio et al., 2018b). MFCV_MAX_ was used to determine whether the maximal recruitment of higher-threshold motor units during the initial contraction phase would predict rapid and peak force production across the investigated groups (Del Vecchio, 2023). The analyses were performed in MATLAB (MathWorks Inc., Natick, MA, USA).

The absolute variability between the two sessions (i.e., day-to-day variability) was calculated for each participant as the percentage absolute difference between the two measurements for the assessed metrics (i.e., MVF, impulse, time-locked RFD, and MFCV_MAX_) using the following formula: D-D Var (%) =|X1-X2|/[(X1-X2)/2]*100. This yields the absolute percent difference, as indicated by the magnitude of the change between sessions relative to the mean of the two values (Bland and Altman, 1986, 1996).

### 2.6 Statistical analysis

The same set of participants was assessed by two distinct grouping approaches: (*a*) according to biological sex (M – F) and (*b*) according to their training history (ST – RA). Therefore, two distinct analyses were performed for each.

The Shapiro-Wilk test was conducted to assess the normality of the data distribution, confirming that all data were normally distributed. Independent-samples t-tests were used to compare anthropometric measures and IPAQ scores between the two groups. Differences in MVF, impulse, MFCV_MAX_, CC from MFCV-estimation, time-locked RFD, t-RFD_MAX_, and day-to-day variability metrics were examined using generalized mixed-effects models (analysis 1: *group*, males-females; *time*, session I -session II; analysis 2: *group*, ST-RA; *time*, session I -session II) and participant ID included as the clustering variable [e.g., MFCV_MAX_ ~ *group* x *time* + (1 | participant ID)]. This approach was used to account for repeated measurements within subjects and preserve inter-individual variability by modeling subject ID as a random effect (Yu et al., 2022; Wilkinson et al., 2023). A Gamma distribution and a Log link function were used to account for positively skewed data (Ng and Cribbie, 2017; Nuccio et al., 2024). RFD0-X_MAX_ and impulse were assessed as both absolute and *relative* (% MVF) metrics. For each dependent variable, a separate generalized mixed-effects model was fitted. Holm–Bonferroni correction was applied within each model to the family of fixed-effect tests (group, time, and group × time). When a significant main effect or interaction was detected, multiplicity correction was applied to the corresponding follow-up contrasts within that same model.

Pearson product-moment CC was used to assess the linear relation between force parameters and MFCV_MAX_ for each cohort, and the coefficient of determination (R^2^) was used as an index of prediction power (Lecce et al., 2025b). The strength of the association was interpreted as follows: 0-0.1, *very weak*; 0.1-0.3, *weak*; 0.3-0.5, *moderate*; 0.5-0.7, *strong*; 0.7-1.0, *very strong* (Ingene and Weisberg, 1981). Differences in regression slopes were assessed by linear regression with a group × covariate interaction and tested using an extra-sum-of-squares F-test (Corotto, 2023). Estimated marginal mean differences together with their 95% confidence intervals were used to quantify the magnitude and precision of statistically significant effects.

Statistical analyses were completed using SPSS, version 23.0 (IBM Corp., Armonk, NY, USA) and Jamovi 2.3.28 (The jamovi project, Sydney, Australia). A *p*-value of < 0.05 was considered statistically significant. The full statistical report is available as **Supplementary Material**.

## 3 Results

### 3.1 BMI, age, and IPAQ score

No differences in body mass index, age, or IPAQ score were observed between participants grouped as ST and RA, or between males and females (*p* > 0.05). The details of these comparisons are presented in **Table 1**.

**Table 1:** Anthropometric characteristics and IPAQ score.

Variables	M	F	*p*-value
Age (years)	23.2 ± 3.8	22.5 ± 2.4	0.691
BMI (kg m^-2^)	24.7 ± 3.9	24.3 ± 2.4	0.732
IPAQ (MET min week^-1^)	2782 ± 1480	2804 ± 1903	0.975

Variables	ST	RA	*p*-value
Age (years)	23.4 ± 2.1	25.4 ± 3.9	0.136
BMI (kg m^-2^)	23.0 ± 3.6	22.9 ± 2.8	0.948
IPAQ (MET min week^-1^)	3425 ± 1848	2267 ± 1319	0.106

F, female group; M, male group; RA, recreationally active group; ST, strength-trained group. Data are presented as mean ± SD.

### 3.2 MVF, impulse, RFD, t-RFD_MAX_

**Figure 2:**
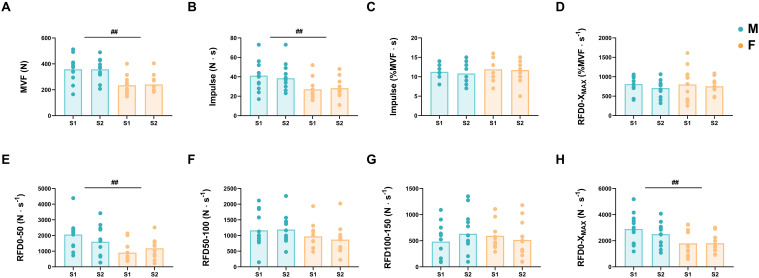
Sex-based comparisons of mechanical parameters. Color-coded bar plots with individual values are displayed for sex-based comparisons across the first (S1) and the second sessions (S2) for the analyzed mechanical parameters. In particular, within-between comparisons for MVF **(A)**, impulse **(B)**, relative impulse **(C)**, relative RFD0- X_MAX_ **(D)**, RFD0-50 **(E)**, RFD50-100 **(F)**, RFD100-150 **(G)**, and RFD0- X_MAX_ **(H) **are presented. ^##^*p* ≤ 0.01 (*group effect*).

When comparing according to sex, a significant group effect was observed for MVF (χ² (1) = 24.31, *p* < 0.001), with higher values in the M group (Δ-MVF = 120 N [73, 168], approximately 33%). A significant group effect was also found for impulse (χ² (1) = 9.62, *p* = 0.002), which was higher in the M group (Δ-impulse = 12.1 N·s [4.5, 19.7]). For RFD, a significant group effect was observed only for RFD0-50 (χ² (1) = 10.21, p = 0.001) and RFD0-XMAX (χ² (1) = 9.84, *p* = 0.002), both of which were higher in the M group (Δ-RFD0-50 = 788 N·s^−1^ [294, 1281]; Δ-RFD0-XMAX = 904 N· s^−1^ [339, 1469]). No significant group × time interactions were observed for RFD50–100 or RFD100-150 (*p* > 0.05). When values were normalized to MVF, no significant group × time interactions were observed for impulse or RFD (*p* > 0.05). These comparisons are presented in **Figure 2**.

**Figure 3:**
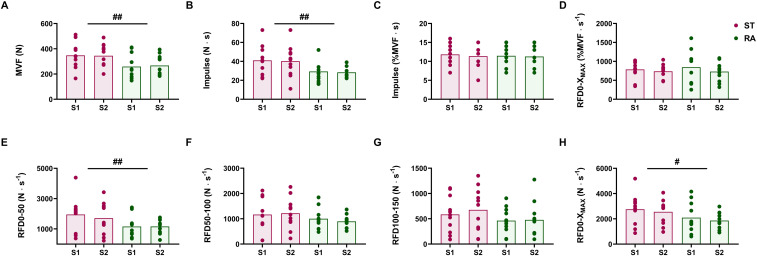
Training history-based comparisons of mechanical parameters. Color-coded bar plots with individual values are displayed for training history-based comparisons across the first (S1) and the second sessions (S2) for the analyzed mechanical parameters. In particular, within-between comparisons for MVF **(A)**, impulse **(B)**, relative impulse **(C)**, relative RFD0- X_MAX_ **(D)**, RFD0-50 **(E)**, RFD50-100 **(F)**, RFD100-150 **(G)**, and RFD0- X_MAX_ **(H) **are presented. ^#^*p* < 0.05 (*group effect*); ^##^*p* ≤ 0.01 (*group effect*).

When comparing by training history, a significant group effect was observed for MVF (χ²(1) = 8.31, p = 0.004), with higher values in the ST group than in the RA group (Δ-MVF = 83 N [27, 139], approximately 25%). A significant group effect was also found for impulse (χ² (1) = 8.67, *p* = 0.003), which was higher in the ST group (Δ-impulse = 11.8 N·s [3.9, 19.5]). For RFD, a significant group effect was observed only for RFD0-50 (χ² (1) = 6.28, p = 0.012) and RFD0-X_MAX_ (χ² (1) = 4.71, *p* = 0.030), both of which were higher in the ST group (Δ-RFD0-50 = 259 N·s^−1^ [148, 1193]; Δ-RFD0-X_MAX_ = 675 N·s^−1^ [64, 1287]). No significant group × time interactions were observed for RFD50–100 or RFD100-150 (*p* > 0.05). When values were normalized to MVF, no significant group × time interactions were observed for impulse or RFD (*p* > 0.05). These comparisons are presented in **Figure 3**.

The t-RFD_MAX_ differed significantly by training history but not by sex. Specifically, ST participants reached their maximal RFD earlier than RA participants (ST: 78.3 ± 18.2 ms; RA: 112.4 ± 24.6 ms; χ² (1) = 6.74, *p* = 0.009). No significant difference was observed between males and females (M: 94.1 ± 27.5 ms; F: 96.8 ± 30.9 ms; *p* > 0.05). These data indicate that the earlier attainment of peak RFD in ST individuals accompanies their higher absolute RFD0-X_MAX_.

### 3.3 MFCV_MAX_ and CC

**Figure 4:**
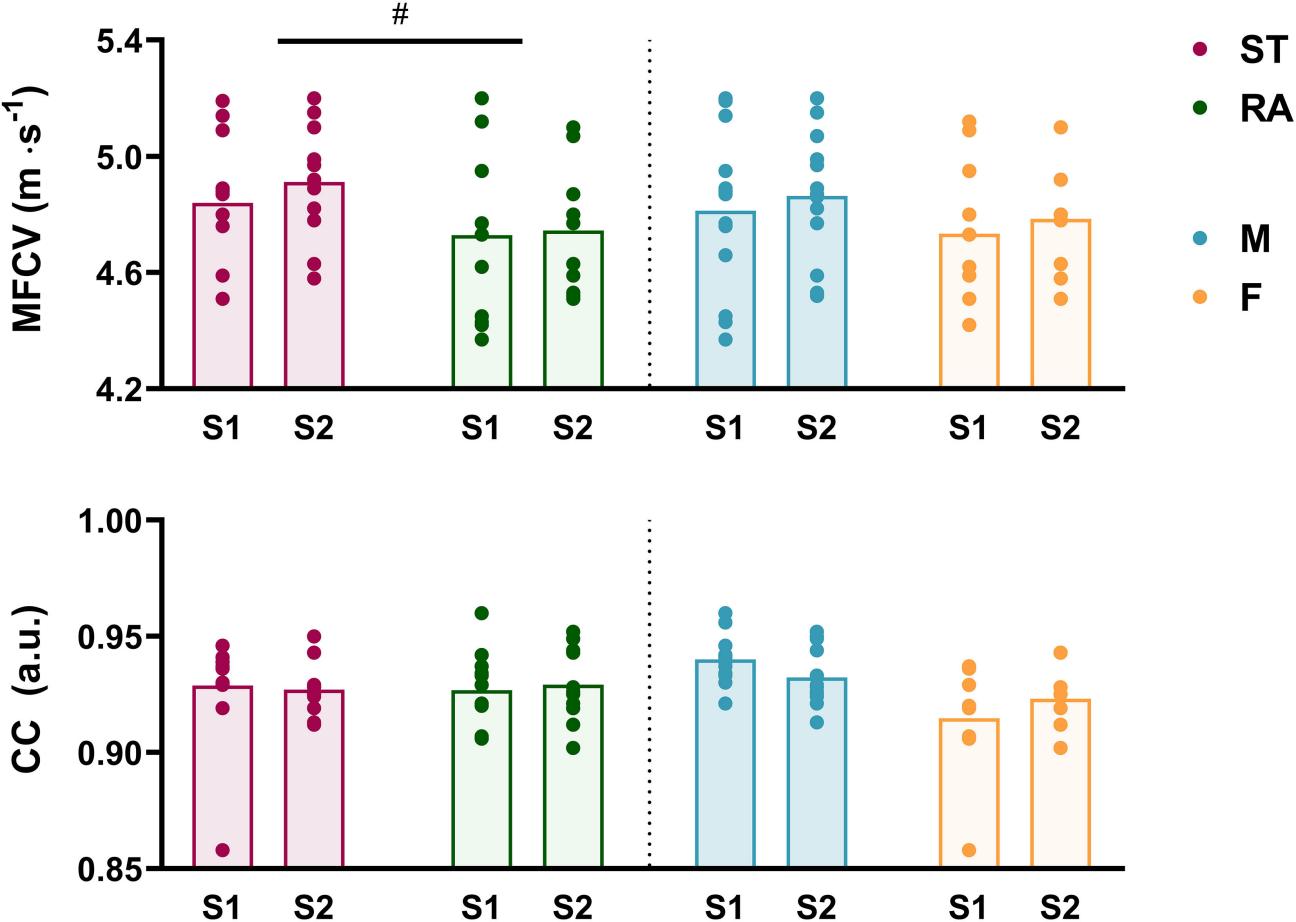
MFCV_MAX_ comparisons according to sex and training history. Color-coded bar plots with individual values are displayed for sex-based and training history-based comparisons across the first (S1) and the second sessions (S2) for MFCV_MAX_ **(A)** and CC **(B)** results. ^#^*p* < 0.05 (*group effect*).

No significant interactions were observed in MFCV_MAX_ when comparing M and F groups (*p* > 0.05). By contrast, a significant *group* effect was found in MFCV_MAX_ when comparing ST and RA (χ² (1) = 3.98, *p* = 0.046), with the ST group showing higher values (Δ-MFCV_MAX_ = 0.14 m·s^−1^ [0.01, 0.27]). No significant group × time interactions were found for CC (*p* > 0.05). The mentioned results are presented in **Figure 4**.

### 3.4 Day-to-day variability

**Figure 5:**
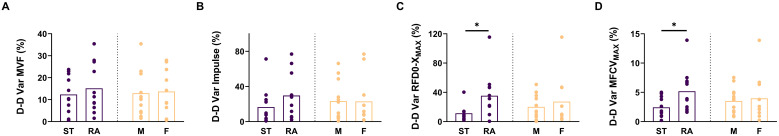
Day-to-day variability of neuromuscular metrics. Color-coded bar plots with individual values are displayed for sex-based and training history-based comparisons for the absolute day-to-day variability (D-D Var) in MVF **(A)**, impulse **(B)**, RFD0- X_MAX_ **(C)**, and MFCV_MAX_ **(D)**. *p < 0.05.

No significant interactions were observed when the sample was examined according to sex (*p* > 0.05). A statistically significant *group* effect was found for the variability in RFD0- X_MAX_ (χ² (1) = 5.74, *p* = 0.017) and MFCV_MAX_ (χ² (1) = 5.01, *p* = 0.025), both lower in ST compared to RA group (Δ-vRFD0- X_MAX_ = 24.0% [3.2, 44.9]; Δ-vMFCV_MAX_ = 2.8% [0.2, 5.3]). No significant interactions have been observed for the variability in the other absolute or relative parameters (*p* > 0.05). The above-mentioned results are presented in **Figure 5**.

### 3.5 Interaction between MFCV and mechanical parameters

**Figure 6:**
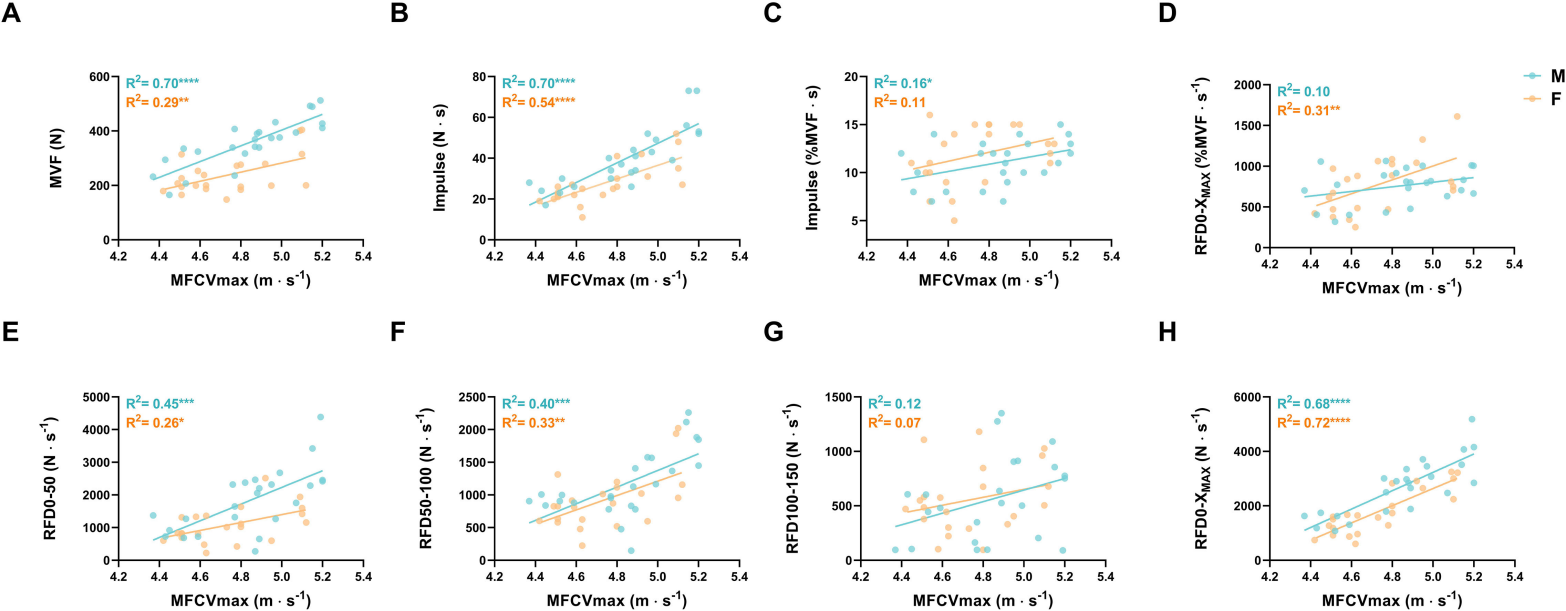
M-F associations between MFCV_MAX_ and mechanical parameters. Color-coded scatter plots with regression lines are presented for the associations between MVF **(A)**, impulse (absolute, **B**; relative, **C**), maximal RFD (relative, **D**; absolute **H**) early RFD **(E, F)**, and late RFD **(G)** against the MFCV_MAX_ between males and females. Color coded R^2^ are reported for each plot. *p < 0.05; **p < 0.01; ***p < 0.001; ****p < 0.0001.

**Figure 7:**
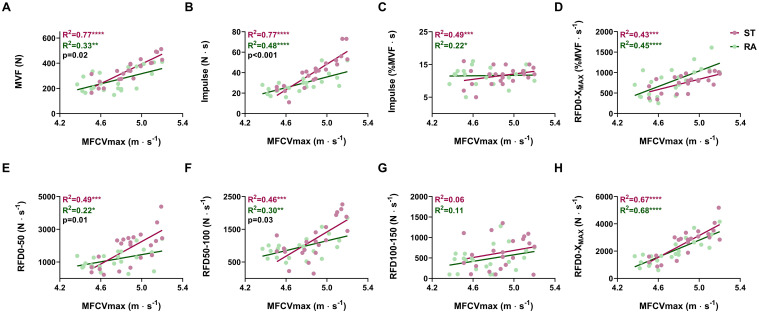
ST-RA associations between MFCVMAX and mechanical parameters. Color-coded scatter plots with regression lines are presented for the associations between MVF **(A)**, impulse (absolute, **B**; relative, **C**), maximal RFD (relative, **D**; absolute **H**) early RFD **(E, F)**, and late RFD **(G)** against the MFCV_MAX_ between ST and RA individuals. Only for significant slope interaction, the *p*-value of comparison is reported within the plots. *p < 0.05; **p < 0.01; ***p < 0.001; ****p < 0.0001.

To address the primary aim of the study, we examined the associations between MFCV_MAX_ and mechanical parameters (MVF, impulse, and RFD), as well as their day-to-day variability, to determine whether MFCV_MAX_ predicts force production capacity and its consistency across groups (**Table 2**; **Figures 6**, **7**). MFCV_MAX_ was significantly associated with MVF and impulse in both sex-based and training history-based comparisons. Similarly, RFD0–50 and RFD0-X_MAX_ were consistently associated with MFCV_MAX_ across groups, whereas no significant associations were observed for later-phase RFD (**Table 2**).

**Table 2:** Associations between MFCV and mechanical variables.

Interaction	M	F	ST	RA
MFCV_MAX_ - MVF	*R²* = 0.71 *p* < 0.0001	*R²* = 0.29 *p* = 0.009	*R²* = 0.77 *p* < 0.0001	*R²* = 0.33 *p* = 0.005
MFCV_MAX_ - Impulse	*R²* = 0.70 *p* < 0.0001	*R²* = 0.54 *p* < 0.0001	*R²* = 0.77 *p* < 0.0001	*R²* = 0.48 *p* = 0.0004
MFCV_MAX_ - %-Impulse	*R²* = 0.16 *p* = 0.04	n.s.	n.s.	n.s.
MFCV_MAX_ - RFD0–50	*R²* = 0.45 *p* = 0.0003	*R²* = 0.26 *p* = 0.01	*R²* = 0.49 *p* = 0.0003	*R²* = 0.22 *p* = 0.02
MFCV_MAX_ - RFD50-100	*R²* = 0.40 *p* = 0.0008	*R²* = 0.33 *p* = 0.005	*R²* = 0.47 *p* = 0.0004	*R²* = 0.30 *p* = 0.007
MFCV_MAX_ - RFD100-150	n.s.	n.s.	n.s.	n.s.
MFCV_MAX_ - RFD0-X_MAX_	*R²* = 0.68 *p* < 0.0001	*R²* = 0.72 *p* < 0.0001	*R²* = 0.67 *p* < 0.0001	*R²* = 0.68 *p* < 0.0001
MFCV_MAX_ - %-RFD0-X_MAX_	n.s.	*R²* = 0.31 *p* = 0.006	*R²* = 0.42 *p* = 0.001	*R²* = 0.45 *p* = 0.0006

F, female group; M, male group; MFCV_MAX_, maximal muscle fiber conduction velocity; RA, recreationally active group; RFD, rate of force development; ST, strength-trained group.

**Figure 8:**
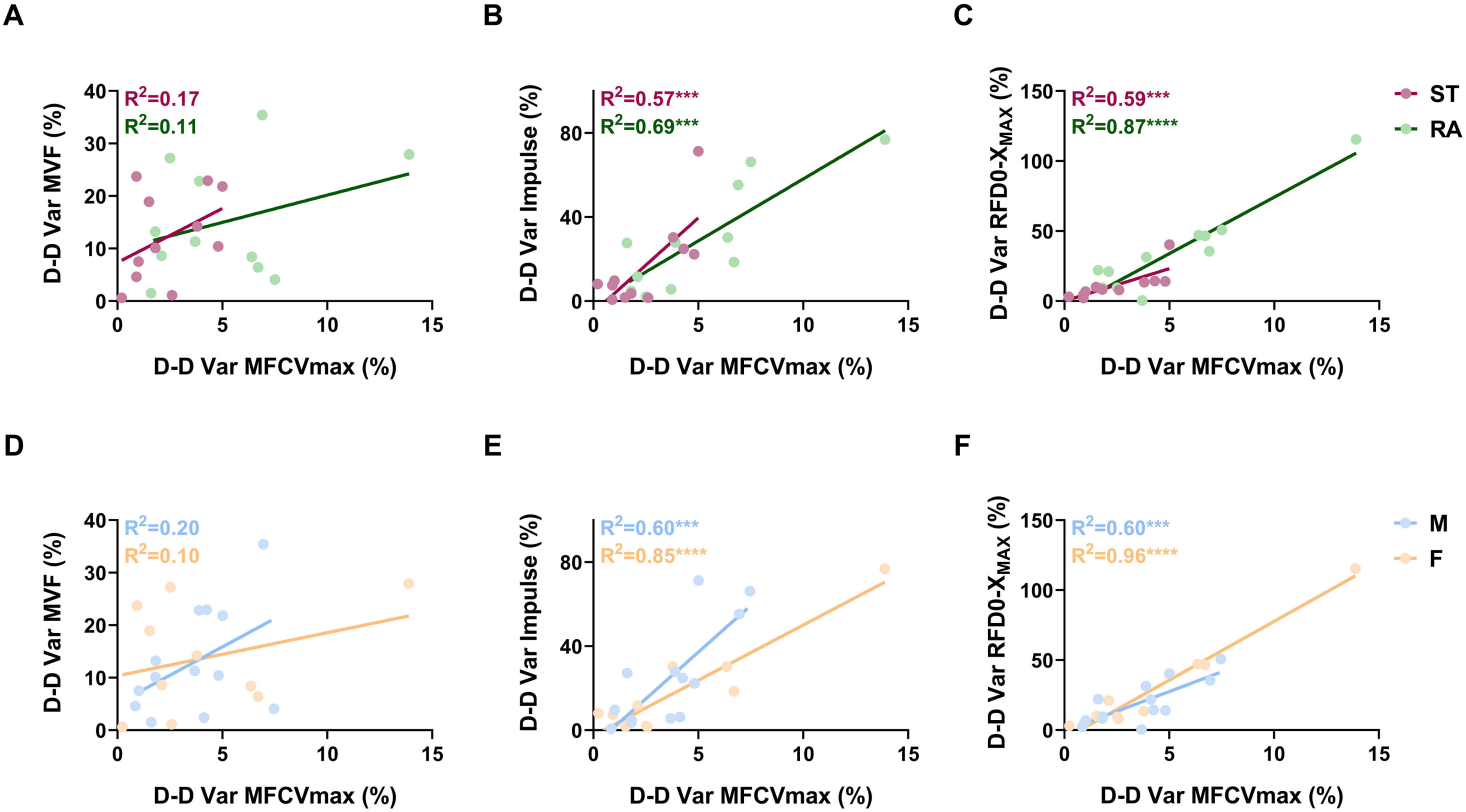
Associations between day-to-day variability in MFCV_MAX_, impulse, and RFD0-X_MAX_. Color-coded scatter plots with regression lines are presented for the associations between the variability between MVF **(A)**, impulse **(B)**, and maximal RFD **(C)** against the variability in MFCV_MAX_ were presented for the comparisons according to the training status. Color-coded scatter plots with regression lines are presented for the associations between the variability between MVF **(D)**, impulse **(E)**, and maximal RFD **(F)** against the variability in MFCV_MAX_ were also presented for comparisons according to biological sex. Color-coded R^2^ are reported for each plot. ***p < 0.001; ****p < 0.0001.

**Figure 9:**
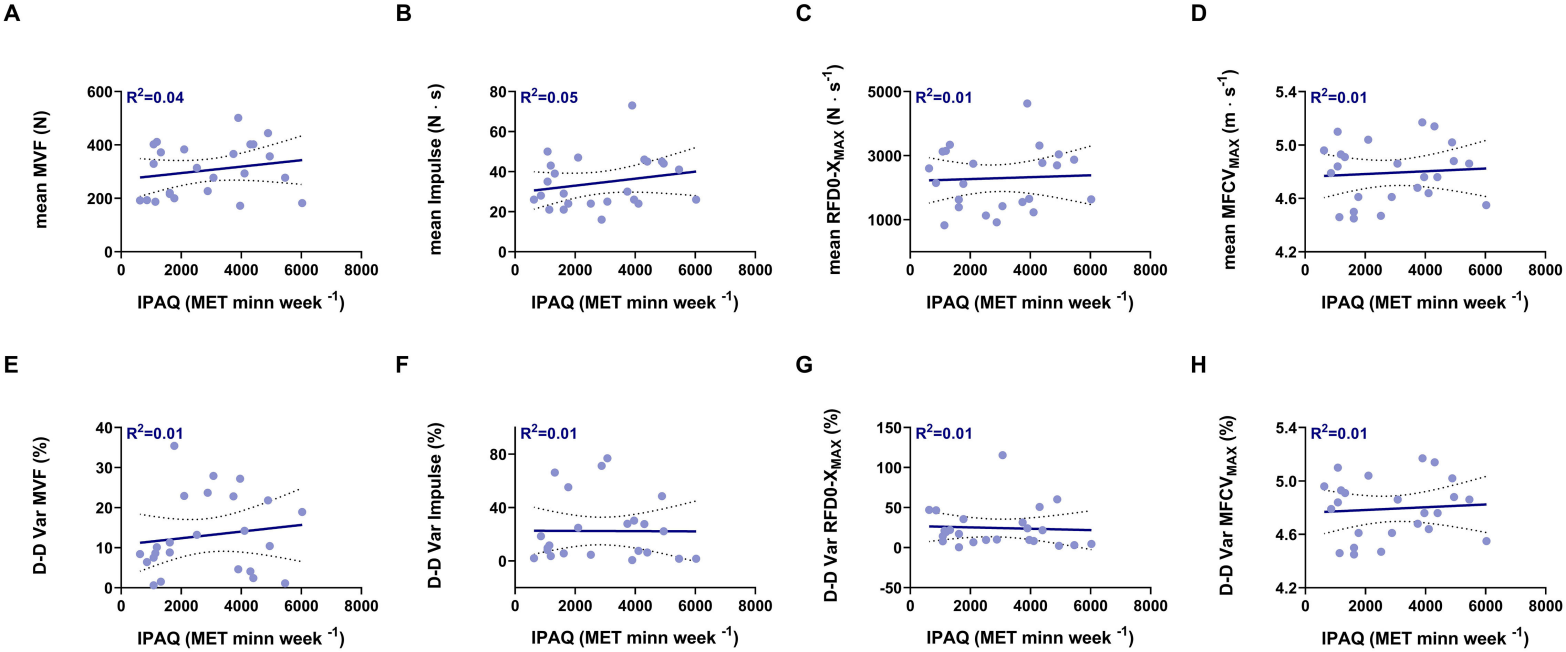
Associations between IPAQ-score, neuromuscular metrics and their variability. Scatter plots display the regressions between IPAQ score and MVF **(A)**, impulse **(B)**, RFD0-X_MAX_ **(C)**, and MFCV_MAX_, **(D)** as well as their variability **(E–H)**. The sample reported includes the whole sample without group discrimination to examine the impact of the general level of habitual training on the mentioned variables.

We further examined whether variability in MFCV_MAX_ predicts variability in mechanical variables (**Table 3**; **Figure 8**). No significant associations were observed between MVF and MFCV_MAX_ variability. However, variability in impulse and RFD0-X_MAX_ was significantly associated with variability in MFCV_MAX_ across both sex-related and training-related groups. No significant associations were found between IPAQ score and neuromuscular variables or their variability (p > 0.05; **Figure 9**).

**Table 3:** Associations between day-to-day variability in MFCV_MAX_ and mechanical variables.

Interaction	M	F	ST	RA
MFCV_MAX_ - MVF	n.s	n.s	n.s	n.s
MFCV_MAX_ - Impulse	R² = 0.60 p = 0.001	R² = 0.85 p = 0.0001	R² = 0.57 p = 0.007	R² = 0.69 p = 0.001
MFCV_MAX_ - RFD0-X_MAX_	R² = 0.60 p = 0.001	R² = 0.96 p < 0.0001	R² = 0.59 p = 0.005	R² = 0.87 p < 0.0001

F, female group; M, male group; MFCV_MAX_, maximal muscle fiber conduction velocity; RA, recreationally active group; RFD, rate of force development; ST, strength-trained group.

## 4 Discussion

The present study examined whether biological sex and training history influence maximal and rapid force production and their day-to-day variability. Both sex and training history affected absolute MVF and rapid-force metrics, but differences in RFD and impulse disappeared after normalizing for MVF. MFCV_MAX_ during initial contraction phases differed by training history but not by sex. Importantly, RFD showed significant day-to-day variability only when comparing ST and RA groups, and this variability was significantly correlated with the relative variability in MFCV_MAX_. Because MFCV_MAX_ increases with the size of recruited motor units, these findings suggest that (*a*) sex effects on rapid force mainly reflect baseline force disparities rather than differences in early motor-unit recruitment, (*b*) training-history effects on rapid force depend on a combined effect of baseline force disparities and distinct motor-unit recruitment during the initial contraction phase, and (*c*) consistent rapid force production across days differs primarily on stable recruitment of similarly sized motor units in the early contraction phase, a characteristic influenced by training history and not by biological sex.

In line with our hypothesis, males displayed higher absolute maximal force (MVF), impulse, and both maximal (RFD0-X_MAX_) and early RFD (RFD0-50) than females. By contrast, we did not observe sex effects on later phase RFD. This discrepancy with previous reports (Hannah et al., 2012; Salvaggio et al., 2025) may reflect differences in sample characteristics, as the present cohort included participants balanced across training backgrounds, whereas earlier studies examined individuals with only moderate physical activity levels. The presence of sex effects on early RFD, which underpin inter-individual differences in absolute force production across a wide range of MVF (Del Vecchio et al., 2018b), indicates that the influence of biological sex on rapid force capacity is primarily associated with maximal force and the early rate of force change.

A methodological consideration concerns the use of individualized time windows to compute RFD0-X_MAX_. ST participants reached their peak RFD approximately 34 ms earlier than RA participants. This systematic difference does not invalidate between-group comparisons of RFD0-X_MAX_; rather, it provides additional physiological insight. The earlier peak in ST individuals is consistent with their higher MFCV_MAX_ and faster recruitment of larger motor units during the initial contraction phase (Del Vecchio et al., 2018b). Moreover, this pattern was preserved using individualized windows, and a comparable pattern of group differences was observed in the time-locked RFD measures (e.g., RFD0-50), which are immune to this concern. Thus, the use of individualized peak detection captures the true maximal neural-mechanical capacity of each participant without biasing the primary conclusions. This approach is consistent with prior work demonstrating that the key neural determinants of RFD are best revealed by individualized, rather than fixed, temporal analyses (Del Vecchio et al., 2019).

Training history also influenced maximal and rapid force characteristics, with ST individuals exhibiting greater MVF, impulse, RFD0–50 and RFD0-X_MAX_ than RA participants, consistent with previous reports (Orssatto et al., 2020; Balshaw et al., 2022; Škarabot et al., 2024). In contrast, RFD during the later phases of contraction did not differ between groups. These results suggest that chronically strength-trained individuals reach higher peak forces and absolute maximal RFD by generating much of their force in the very initial phase of a ballistic contraction, as previously observed (Del Vecchio et al., 2018b). Therefore, much of the between-group differences in maximal RFD are dictated by greater early-phase force production in the ST group, and once contractions progress into later phases, the *rate of change* of force becomes comparable between ST and RA participants (Maffiuletti et al., 2016; Dideriksen et al., 2020; Kozinc et al., 2022; Del Vecchio, 2023).

Together, these findings indicate that both biological sex and training history substantially influence maximal strength and absolute indices of rapid force capacity.

When impulse and RFD0-X_MAX_ were normalized to MVF, however, the values were comparable across and within both cohorts. This similarity reflects comparable relative force-time profiles and suggests that group effects on absolute impulse and maximal RFD are primarily attributable to baseline differences in force capacity, as indexed by MVF (Folland et al., 2014). This interpretation is further supported by the observation that early RFD, which accounts for a large proportion of force increase during explosive contractions (Maffiuletti et al., 2016; Del Vecchio et al., 2018b), was also similar between groups after normalization to MVF. Accordingly, neither biological sex nor training history appears to influence the ability to rapidly express the available force capacity, consistent with previous sex-based (Grootenhuis et al., 2025; Salvaggio et al., 2025) and training history-based comparisons (Del Vecchio et al., 2018b; Škarabot et al., 2024).

The analysis of neuromuscular properties was restricted to measures exhibiting excellent reliability (cross-correlation coefficient ≥ 0.85), consistent with expectations for multichannel EMG-based estimations and supported by extensive prior evidence (Farina et al., 2001, 2004a, 2004b; Del Vecchio et al., 2018b). In the present study, MFCV_MAX_ ranged from approximately 3.3 to 5.3 m·s^−1^, in agreement with previous reports obtained from the elbow flexor muscles (Zwarts and Arendt-Nielsen, 1988; Farina et al., 2004b; Bazzucchi et al., 2011; Del Vecchio et al., 2018b).

To our knowledge, this is the first study to assess MFCV_MAX_ during the initial phase of maximal ballistic contractions and to compare these metrics between males and females concurrently. Previous findings investigating MFCV under electrical stimulation indicate a faster conduction velocity in males than in females, which the authors attribute to greater muscle fiber size in males (Mase et al., 2006; Kouyoumdjian and Graca, 2023). In contrast, prior evidence based on voluntary contractions indicates comparable MFCV_MAX_ during MVCs (Inglis et al., 2017). In agreement with findings on voluntary contractions, our results suggest that MFCV_MAX_ during the early contraction phases of voluntary ballistic contractions is not different between sexes when training status is matched. This observation suggests similar neural control during rapid voluntary contractions and aligns with recent evidence indicating comparable motor-unit discharge behavior between males and females during explosive tasks (Grootenhuis et al., 2025).

Given that maximal motoneuron discharge rate is closely linked to maximal RFD (Desmedt and Godaux, 1978; Duchateau and Baudry, 2014; Del Vecchio et al., 2019; Škarabot et al., 2024) and that recruitment threshold substantially influences discharge rate at a given absolute force (Duchateau and Baudry, 2014; Lecce et al., 2026), our findings imply that the rate of motor-unit recruitment may be similar between sexes. Recruitment rate is considered a key determinant of maximal RFD (Maffiuletti et al., 2016; Del Vecchio, 2023). Accordingly, our results indicate no differences in recruitment of high-threshold motor units between sexes, supporting the prevailing view that sex-related differences in rapid force production arise primarily from differences in contractile properties rather than from neural control during ballistic contractions (Salvaggio et al., 2025).

By contrast, the ST group exhibited a higher MFCV_MAX_, which was strongly associated with their greater RFD and MVF and is consistent with prior reports (Methenitis et al., 2016; Del Vecchio et al., 2018b). Rapid force production is considerably influenced by the early activation of larger motor units, which innervate numerous muscle fibers and produce greater mechanical output (Heckman and Enoka, 2012; Dideriksen and Farina, 2013). Accordingly, we found no relationship between MFCV_MAX_ and later-phase RFD, supporting the evidence that contractile properties rather than neuromuscular control are the predominant determinant in this later interval (Maffiuletti et al., 2016; D’Emanuele et al., 2023).

The greater MFCV_MAX_ observed in chronically strength-trained individuals may also reflect larger muscle-fiber diameter (Hakansson, 1956; Andreassen and Arendt-Nielsen, 1987; Del Vecchio et al., 2018c; Casolo et al., 2023), a predictable effect of long-term hypertrophic adaptation (Methenitis et al., 2016; Del Vecchio et al., 2018b). Nevertheless, the higher maximal RFD and impulse observed in ST individuals were also accompanied by higher MFCV_MAX_, suggesting that their greater capacity for rapid force production stems from both greater absolute muscle force and a more consistent early recruitment of high-threshold motor units (Del Vecchio et al., 2018b; Škarabot et al., 2024), as confirmed by the strong associations between these variables.

Habitual physical activity, as assessed by IPAQ, was not associated with neuromuscular performance or with day-to-day variability. This result supports the view that sheer activity volume, in the absence of a task-specific stimulus, is insufficient to elicit distinct neuromuscular adaptations (Stone et al., 2022; Del Vecchio et al., 2024; Lecce et al., 2024b). Consequently, self-reported habitual activity has limited predictive value for outcomes such as maximal force, rapid-force capacity and their consistency, and should be interpreted alongside objective, task-specific measures (Prince et al., 2008; Leblanc et al., 2015; Silsbury et al., 2015; Rostron et al., 2021).

When training history was matched, we found no sex-related effects on the day-to-day variability of maximal or rapid force production. Previous reports are mixed, with some suggesting greater consistency in females (Luger et al., 2020) and others showing comparable variability between sexes (Dutra et al., 2023). In our sample, similar variability in MVF, RFD, and impulse across sexes was paralleled by comparable variability in MFCV_MAX_. On the other hand, ST showed significantly lower day-to-day variability in maximal RFD than RA individuals, as expected from prior investigations (Levernier and Laffaye, 2021). Moreover, larger day-to-day fluctuations in mechanical outputs co-occurred with greater variability in MFCV_MAX_. Together, these observations suggest that chronically strength-trained individuals, who exhibited higher MFCV_MAX_, also showed more stable recruitment of high-threshold motor units across sessions. This more stable neuromuscular consistency likely underpins the greater consistency of rapid force production observed in ST compared with RA individuals.

In conclusion, we demonstrated that biological sex and training history affected maximal and rapid force production, as well as their day-to-day variability. We found that sex influences absolute MVF, RFD, and impulse, but not MFCV_MAX_, suggesting that sex differences in rapid force are driven by baseline mechanical capacity rather than early motor-unit recruitment. In contrast, training history affected MVF, RFD, impulse, and MFCV_MAX_, implying that strength training produces both contractile adaptations and enhanced recruitment of higher-threshold motor units during the initial contraction phase. Importantly, neither sex nor training history was shown to affect the capacity to rapidly generate available force, as evidenced by similar relative impulse and RFD. Finally, day-to-day variability differed according to training history, whereas no statistically significant sex-related differences were detected in the present cohort, likely dependent on the greater ability to consistently recruit larger motor units early in contraction in ST individuals. In addition to the functional implications for the study of human rapid force, the study also presents a methodology that may be employed to assess neural strategies of muscle control and their variability in health, training, and clinical settings.

## 5 Limitations and future directions

A limitation of the present study is that impulse was analyzed without direct assessment of segmental inertia or limb mass distribution. Although BMI did not differ between groups, absolute impulse is a force-time integral and may therefore be modulated not only by neural drive and contractile behavior, but also by anthropometric and inertial properties of the moving segment. Accordingly, between-group differences in absolute impulse cannot be attributed exclusively to neuromuscular factors. Additionally, day-to-day variability was estimated from only two experimental sessions. Although this approach is commonly used in reliability studies, it may not fully capture biological variability across multiple days or weeks. Consequently, the present estimates should be interpreted as short-term between-session variability rather than a comprehensive representation of long-term neuromuscular fluctuations. An additional limitation concerns the sample size available for sex-specific analyses. Although the study was adequately powered for the primary outcome (i.e., RFD), the number of males (n = 12) and females (n = 10) may not have been sufficient to detect small sex-related differences in day-to-day variability measures. Consequently, the absence of statistically significant sex effects should be interpreted cautiously, as a type II error cannot be excluded. Future studies incorporating direct measures of limb inertia and alternative temporal normalization approaches are warranted to clarify how these factors interact with rapid force production in humans. Also, future investigations employing larger and more balanced cohorts are warranted to confirm whether biological sex influences the stability of rapid force production across repeated assessments.

## Data Availability

The original contributions presented in the study are included in the article/**Supplementary Material**. Further inquiries can be directed to the corresponding author.
